# Sex-specific associations between diabetes and dementia: the role of age at onset of disease, insulin use and complications

**DOI:** 10.1186/s13293-023-00491-1

**Published:** 2023-02-20

**Authors:** Chunmiao Zhou, Caiyun Dong, Ziwei Xie, Wenting Hao, Chunying Fu, Huizi Sun, Dongshan Zhu

**Affiliations:** 1 Department of Epidemiology, School of Public Health, Cheeloo College of Medicine, Shandong University, 44 Wenhuaxi Road, Jinan, 250012 Shandong China; 2 Centre for Health Management and Policy Research, School of Public Health, , Cheeloo College of Medicine, Shandong University, Jinan, 250012 China; 3 NHC Key Lab of Health Economics and Policy Research (Shandong University), Jinan, 250012 China

**Keywords:** Type 2 diabetes, Dementia, Sex, Age at onset of diabetes, Insulin use, Complications

## Abstract

**Background:**

Whether the association of type 2 diabetes (T2DM) with dementia was differed by sex remains unclear, and the roles of age at onset of disease, insulin use and diabetes’ complications in their association are unknown.

**Methods:**

This study analyzed data of 447 931 participants from the UK Biobank. We used Cox proportional hazards models to estimate sex-specific hazard ratios (HRs) and 95% confidence intervals (CI), and women-to-men ratio of HRs (RHR) for the association between T2DM and incident dementia [all-cause dementia, Alzheimer's disease (AD), and vascular dementia (VD)]. The roles of age at onset of disease, insulin use and diabetes’ complications in their association were also analyzed.

**Results:**

Compared to people with no diabetes at all, people with T2DM had increased risk of all-cause dementia (HR 2.85, 95% CI 2.56–3.17). The HRs between T2DM and AD were higher in women than men, with an RHR (95%CI) of 1.56 (1.20, 2.02). There was a trend that people who experienced T2DM before age 55 had higher risk of VD than those who had T2DM after age 55. In addition, there was a trend that T2DM had higher effect on VD that occurred before age 75 years than events that occurred after age 75. Patients with T2DM using insulin had higher risk of all-cause dementia than those without insulin, with an RHR (95%CI) of 1.54 (1.00–2.37). People with complications had doubled risk of all-cause dementia, AD and VD.

**Conclusions:**

Adopting a sex-sensitive strategy to address the risk of dementia in patients with T2DM is instrumental for a precision medicine approach. Meanwhile, it is warranted to consider patients' age at onset of T2DM, insulin use status and complications conditions.

**Supplementary Information:**

The online version contains supplementary material available at 10.1186/s13293-023-00491-1.

## Highlights

- We evaluated the sex-specific association of T2DM with dementia subtypes, and to examine the roles of age at onset of disease, insulin use and diabetes’ complications in their association.
- Women with T2DM were 1.56 times more likely to experience AD than men.
- Early onset T2DM (younger than age 55 years) might be related to higher risk of VD than later onset T2DM (later than age 55 years).
- Insulin use was linked to 1.5- and 1.9-times higher risk of all-cause dementia and VD, respectively.
- Different types and numbers of complications modulate the risk of dementia in patients with T2DM.

**Supplementary Information:**

The online version contains supplementary material available at 10.1186/s13293-023-00491-1.

## Introduction

With aging and the increase of life expectancy, dementia has become a great global public health challenge [1, 2]. There were over 55 million people living with dementia and the number will reach 78 million by 2030 [3, 4]. Evidence has suggested that the risk of dementia varied by sex [4] and the prevalence of dementia is also sex specific [5]. Almost two-thirds of Alzheimer's disease (AD) patients are women, while vascular dementia (VD) is more common in males than in females [6].

People with type 2 diabetes (T2DM) are more likely to experience dementia [3, 7, 8]. However, the sex-specific association between diabetes and dementia are less well-studied. A review of 14 studies showed that women with T2DM have 19% higher risk of developing VD than men, while the risk of AD did not differ significantly between men and women [9]. Another study showed that women with diabetes are more likely to develop all-cause dementia than men, but not by dementia subtypes [10]. However, other studies observed no interaction between sex and diabetes on dementia [11–13]. Thus, the sex-specific association in the relationship between T2DM and dementia subtypes may need to be further explored. In addition, some studies have shown that complications of T2DM might affect the cognitive aging [14–16], while no study has examined the roles of specific types of diabetes’ complications (such as micro/macro-vascular complications, ketoacidosis etc.) on the association of T2DM with dementia, and whether the roles of these complications differed by sex is unclear. Furthermore, around 30% patients with T2DM need insulin treatment with the disease progression [17]. Evidence has shown that insulin has a potential protective effect on cognitive function [18]. However, a few studies demonstrated that insulin use increased the risk of dementia [7, 19], which might be related to an increased risk of hypoglycemia [20]. How the use of insulin therapy moderates the link between T2DM and dementia and whether there is a sex difference remains unclear.

The aim of this study is to examine the sex-specific association of T2DM with dementia subtypes, and to examine the roles of age at onset of disease, insulin use and diabetes’ complications in their association. We hypothesized that there was a sex-specific association between diabetes and dementia, and early onset T2DM, insulin use and diabetes’ complications might strengthen the risk between diabetes and dementia.

## Methods

### Study design and participants

We used data from the UK Biobank. The UK Biobank is a large population-based prospective cohort study that recruited over 502 000 participants aged 40–70 years from 2006 to 2010 [21]. Written informed consent was obtained for collection of questionnaire and biological data. All participants were linked to hospital data and national death registries from England, Scotland and Wales to determine the date of the first diagnosis of dementia after the baseline assessment. UK Biobank received ethical approval from the UK National Health Service’s National Research Ethics Service (ref 11/NW/0382). This research was conducted under UK Biobank application number 68369. A prospective design was adopted based on participants with no dementia at baseline, and if a participant had dementia during follow-up and also experienced T2DM, his/her diagnosis of T2DM had to be in advance of dementia. Finally, 447 931 people were included in this prospective analysis (Additional file 1: Figure S1). This study is reported as per the Strengthening the Reporting of Observational Studies in Epidemiology (STROBE) guidelines (Additional file 8: Appendix).

### Exposure and outcome

The exposure variable was the occurrence of T2DM. Physician-diagnosed T2DM was ascertained from linkage data to primary care, hospital admission and death register records. The International Classification of Diseases 10th Edition (ICD-10) code E11 and ICD-9 code 250 were used to identify participants with T2DM. T2DM-related complication types were defined by the ICD-10 codes E11·0-E11·5 and ICD-9 code 250. Insulin use status was self-reported. The outcome variable was incident all-cause dementia, including dementia subtypes of AD and VD. The ICD-10 codes F00, F01, G30, and ICD-9 code 290·1 were used to identify participants with all-cause dementia if one or more of these codes were recorded as a primary or secondary diagnosis in the health records. Incident AD was defined by ICD-10 codes F00, G30 and ICD-9 code 290·1. Incident VD was defined by ICD-10 code F01. Outcome adjudication for incident dementia was conducted by the UK Biobank Outcome Adjudication team. Age onset of T2DM or dementia was defined as the first occurrence of event and was sourced from primary care, hospital admissions and mortality data. Specific field IDs or ICD codes for T2DM, insulin use, complication status, and dementia types were listed in Additional file 2: Table S1.

### Covariates

We included the following factors in the analyses as covariates according to evidence from previous studies [22–24]: age at last follow-up, race/ethnicity, years of education, income level, smoking status, physical activity strength, number of leisure activities, BMI, hypertension status, APOE4 allele status, HbA1c level, cardiovascular diseases (CVD) status and depressive status. Race/ethnicity was categorized as white and non-white. Years of education was categorized as ≤ 10, 11–12, and > 12 years. Income level was divided into four categories of level 1 (Less than £18,000), level 2 (£18,000 to £30,999), level 3 (£31,000 to £51,999) and level 4 (greater than 52,000). Smoking status was categorized as current, former, or never smokers. Physical activity level was categorized as light (< 600 metabolic equivalent (MET)-min/week), moderate (600 to < 3000 MET-min/week) and high (≥ 3000 MET-min/week) based on standard scoring criteria. Number of leisure activities was categorized as none, one, and two or more. BMI was calculated as weight in kilograms divided by the square of height in meters and categorized according to the World Health Organization criteria as < 18.5 kg/m^2^, 18.5 to 24.9 kg/m^2^, 25 to 29.9 kg/m^2^, and ≥ 30 kg/m^2^. Hypertension status was dichotomized as present or absent based on self-report at baseline. APOE allele status was based on two single nucleotide polymorphisms (SNPs): rs7412 and rs429358. Participants with APOE e4 allele (e3/e4, e4/e4 and occasionally e2/e4 genotypes) were compared with those with the e2/e2, e2/e3 or e3/e3 genotype. HbA1c level was divided into two categories based on the target of less than 7% or not [17]. CVD or depressive status was dichotomized as present or absent based on hospital medical records at baseline.

### Statistical analyses

Baseline characteristics were presented as means and standard deviation (SD) for continuous variables and as percentages (%) for categorical variables. Cox proportional hazards regression models were used to estimate the sex-specific hazard ratios (HR) and 95% confidence intervals (CI) between T2DM and dementia (including all-cause dementia, AD and VD). The interaction term between T2DM and sex was used to obtain the women-to-men ratio of hazard ratios (RHR) for each dementia type and T2DM. The proportional hazards (PH) assumption was tested graphically using a plot of the log cumulative hazard, where the logarithm of time is plotted against the estimated log cumulative hazard. The curves for the two T2DM status (Experienced or not) were approximately parallel, thus the PH assumption was deemed reasonable. Hospital inpatient data and death data were censored on the 30 January 2021 or when death, fatal or non-fatal dementia was recorded. For participants who experienced a dementia, follow-up time was calculated as their age when dementia was diagnosed minus baseline age; for participants without experiencing dementia, follow-up time was defined as their age at last follow-up (censored date) minus baseline age.

To examine whether timing of T2DM occurrence moderate the association between T2DM and dementia, we divided people with T2DM by age at onset of T2DM (< 55 years and ≥ 55 years). We defined “early-onset” diabetes as diabetes diagnosed prior to age 55 years, as 55 years was the median age onset of T2DM in UK Biobank population and our definition was in line with previous studies [25, 26]. In addition, to examine the association between T2DM and timing of dementia, we categorized people with dementia by age when dementia was diagnosed (< 75 and ≥ 75 years). We used age 75 years as the cutoff point to categorize age onset of dementia, as 75 years was the median age of onset of dementia in UK Biobank population and dementia mostly occurs in people over 75 years of age [27]. Furthermore, to investigate whether insulin use moderate the relationship between T2DM and dementia, we divided patients with T2DM into two groups according to whether they used insulin or not. Last, we also analyzed the link between T2DM and dementia by number of complications (0, 1, ≥ 2) and type of complications.

People with no diabetes at all were used as the reference group in all analyses to make the estimates comparable. HRs (95% CI) were adjusted for age at last follow-up, race/ethnicity, years of education, income level, smoking status, physical activity level, number of leisure activities, BMI, hypertension status, HbA1c level, APOE4 allele status CVD status and depressive status.

## Results

### Characteristics of participants (Table 1)

**Table 1:** Characteristics of participants by sex, diabetes and dementia experienced or not, *n* (%)

Characteristics	*N*	Diabetes experienced or not				Dementia experienced or not			
		Female (*n* = 243 659)		Male (*n* = 204 256)		Female (*n* = 243 659)		Male (*n* = 204 256)	
		No	Yes	No	Yes	No	Yes	No	Yes
Age (mean ± SD)		56.1 ± 8.0	60.1 ± 6.8	56.3 ± 8.2	60.7 ± 6.6	56.1 ± 8.0	64.7 ± 4.2	56.5 ± 8.2	64.8 ± 4.2
Race/ethnicity									
White	425,797	224,649 (52.8)	7120 (1.7)	180,917 (42.5)	13,111 (3.1)	230,356 (54.1)	1413 (0.3)	192,513 (45.2)	1515 (0.4)
Non-White	22,118	10,832 (49.0)	1058 (4.8)	8657 (39.1)	1571 (7.1)	11,843 (53.5)	47 (0.2)	10,170 (46.0)	58 (0.3)
Education level (years)									
≤ 10	218,555	113,764 (52.1)	4868 (2.2)	91,471 (41.9)	8452 (3.9)	117,699 (53.9)	933 (0.4)	98,928 (45.3)	995 (0.5)
11–12	54,033	30,368 (56.2)	897 (1.7)	21,219 (39.3)	1549 (2.9)	31,113 (57.6)	152 (0.3)	22,610 (41.8)	158 (0.3)
> 12	175,327	91,349 (52.1)	2413 (1.4)	76,884 (43.9)	4681 (2.7)	93,387 (53.3)	375 (0.2)	81,145 (46.3)	420 (0.2)
Physical activity level									
Light	100,923	53,889 (53.4)	2625 (2.6)	39,997 (39.6)	4412 (4.4)	56,195 (55.7)	319 (0.3)	44,065 (43.7)	344 (0.3)
Moderate	179,909	98,540 (54.8)	3241 (1.8)	72,366 (40.2)	5762 (3.2)	101,174 (56.2)	607 (0.3)	77,521 (43.1)	607 (0.3)
High	167,083	83,052 (49.7)	2312 (1.4)	77,211 (46.2)	4508 (2.7)	84,830 (50.8)	534 (0.3)	81,097 (48.5)	622 (0.4)
Income level (£)									
Less than 18,000	98,892	56,481 (57.1)	2963 (3.0)	35,018 (35.4)	4430 (4.5)	58,780 (59.4)	664 (0.7)	38,813 (39.3)	635 (0.6)
18,000 to 30,999	109,569	59,462 (54.3)	2173 (2.0)	43,997 (40.2)	3937 (3.6)	61,234 (55.9)	401 (0.4)	47,464 (43.3)	470 (0.4)
31,000 to 51,999	116,996	60,475 (51.7)	1664 (1.4)	51,572 (44.1)	3285 (2.8)	61,908 (52.9)	231 (0.2)	54,584 (46.7)	273 (0.2)
Greater than 52,000	122,458	59,063 (48.2)	1378 (1.1)	58,987 (48.2)	3030 (2.5)	60,277 (49.2)	164 (0.1)	61,822 (50.5)	195 (0.2)
No. of leisure activities									
No	125,740	65,661 (52.2)	2894 (2.3)	52,336 (41.6)	4849 (3.9)	68,101 (54.2)	454 (0.4)	56,693 (45.1)	492 (0.4)
One	195,153	100,096 (51.3)	3305 (1.7)	85,159 (43.6)	6593 (3.4)	102,748 (52.7)	653 (0.3)	91,008 (46.6)	744 (0.4)
Two or more	127,022	69,724 (54.9)	1979 (1.6)	52,079 (41.0)	3240 (2.6)	71,350 (56.2)	353 (0.3)	54,982 (43.3)	337 (0.3)
Body mass index (kg/m^2^)									
Underweight < 18.5	2370	1883 (79.5)	12 (0.5)	463 (19.5)	12 (0.5)	1880 (79.3)	15 (0.6)	465 (19.6)	10 (0.4)
Normal [18.5,25.0)	151,122	97,566 (64.6)	865 (0.6)	51,316 (34.0)	1375 (0.9)	97,893 (64.8)	538 (0.4)	52,292 (34.6)	399 (0.3)
Overweight [25.0,30.0)	192,157	87,524 (45.6)	2284 (1.2)	96,754 (50.4)	5595 (2.9)	89,249 (46.5)	559 (0.3)	101,630 (52.9)	719 (0.4)
Obese** ≥ **30	102,266	48,508 (47.4)	5017 (4.9)	41,041 (40.1)	7700 (7.5)	53,177 (52.0)	348 (0.3)	48,296 (47.2)	445 (0.4)
Smoking status									
Never	247,137	140,985 (57.1)	4650 (1.9)	95,912 (38.8)	5590 (2.3)	144,836 (58.6)	799 (0.3)	100,883 (40.8)	619 (0.3)
Past	155,348	74,257 (47.8)	2823 (1.8)	70,918 (45.7)	7350 (4.7)	76,539 (49.3)	541 (0.4)	77,477 (49.9)	791 (0.5)
Current	45,430	20,239 (44.6)	705 (1.6)	22,744 (50.1)	1742 (3.8)	20,824 (45.8)	120 (0.3)	24,323 (53.5)	163 (0.4)
Hypertension status									
No	327,576	182,082 (55.6)	2558 (0.8)	137,733 (42.1)	5203 (1.6)	183,800 (56.1)	840 (0.3)	142,095 (43.4)	841 (0.3)
Yes	120,339	53,399 (44.4)	5620 (4.7)	51,841 (43.1)	9479 (7.9)	58,399 (48.5)	620 (0.5)	60,588 (50.4)	732 (0.6)
APOE									
No APOE4	339,485	177,856 (52.4)	6317 (1.9)	143,782 (42.4)	11,530 (3.4)	183,494 (54.1)	679 (0.2)	154,464 (45.5)	848 (0.3)
One APOE4	99,358	52,835 (53.2)	1716 (1.7)	41,914 (42.2)	2893 (2.9)	53,951 (54.3)	600 (0.6)	44,244 (44.5)	563 (0.6)
Two APOE4	9072	4790 (52.8)	145 (1.6)	3878 (42.8)	259 (2.9)	4754 (52.4)	181 (2.0)	3975 (43.8)	162 (1.8)

MET, metabolic equivalent; APOE, apolipoprotein E

Of the 447 915 people included in this study, 54.40% were female (around 76.5% of women were postmenopausal). The mean (SD) age was 56.3 (8.0) years. The age range of participants was from 40 to 73 years at baseline. After 11.0 (11.0, 12.0)-year follow-up, 3033 participants (48.13% were females) developed dementia. The crude incidence rate of all-cause dementia was 0.53 for women and 0.70 for men, per 1000 person-years, respectively. Compared to individuals who had no dementia, those who experienced dementia were more likely to be less educated, lower income, fewer leisure activities, with hypertension and with two ApoE4 allele. Underweight women had higher proportion of dementia in women than men, while overweight or obese men had higher proportion of dementia than women. Besides, in males, the prevalence of dementia was higher in ever smokers.

### Type 2 diabetes, sex and dementia (Table 2, Additional file 3: Table S2 and Additional file 4: Table S3)

**Table 2:** Sex-specific hazard ratios (HRs) and 95%CIs between type 2 diabetes and dementia subtypes*

	All-cause dementia			Alzheimer's disease			Vascular dementia		
	Dementia events (n)	Events per 1000 person-years	Adjusted HR (95% CI)*	Dementia events (n)	Events per 1000 person-years	Adjusted HR (95% CI)*	Dementia events (n)	Events per 1000 person-years	Adjusted HR (95% CI)*
People with no diabetes at all	2561	0.54	Reference	1918	0.40	Reference	817	0.17	Reference
People with type 2 diabetes									
All patients	562	2.55	2.85 (2.56, 3.17)	322	1.46	2.38 (2.07, 2.73)	302	1.37	3.93 (3.36, 4.59)
Female patients	207	2.51	2.93 (2.59, 3.32)	140	1.69	2.74 (2.24, 3.35)	84	1.02	2.67 (2.04, 3.49)
Male patients	355	2.57	2.70 (2.28, 3.18)	182	1.31	2.15 (1.81, 2.56)	218	1.57	4.73 (3.99, 5.62)
Ratio of HR (Female/Male)			1.19 (0.96, 1.46)			1.56 (1.20, 2.02)			0.89 (0.65, 1.22)

^*^ All HRs were adjusted for age at last follow-up, race/ethnicity, educational years, income level, physical activity level, leisure activities, body mass index (BMI), smoking status, hypertension status and APOE4 allele status

Compared to people with no diabetes at all, people with T2DM were associated with higher risk of all-cause dementia, AD and VD, with HRs (95% CI) of 2.85 (2.56, 3.17), 2.38 (2.07, 2.73), and 3.93 (3.36, 4.59), respectively. After the analyses were stratified by sex, compared to people without diabetes, the HRs (95%CI) between T2DM and AD in women (HR 2.74, 95% CI 2.24–3.35) were higher than the estimates in men (2.15, 1.81–2.56), with an RHR (Female/Male) (95%CI) of 1.56 (1.20, 2.02). No sex difference was observed on the association of T2DM with VD. Similar finding were observed after further adjusted for depressive status (Additional file 3: Table S2). However, after adjusting for CVD status, the HRs (95%CI) between T2DM and all-cause dementia were higher in women (2.59, 2.19–3.06) than men (2.58, 2.27–2.93), with an RHR (95%CI) (Female/Male) of 1.23 (1.00, 1.51) (Additional file 4: Table S3).

### T2DM and age at onset of dementia (Fig. 1 and Additional file 5: Table S4)

**Fig. 1:**
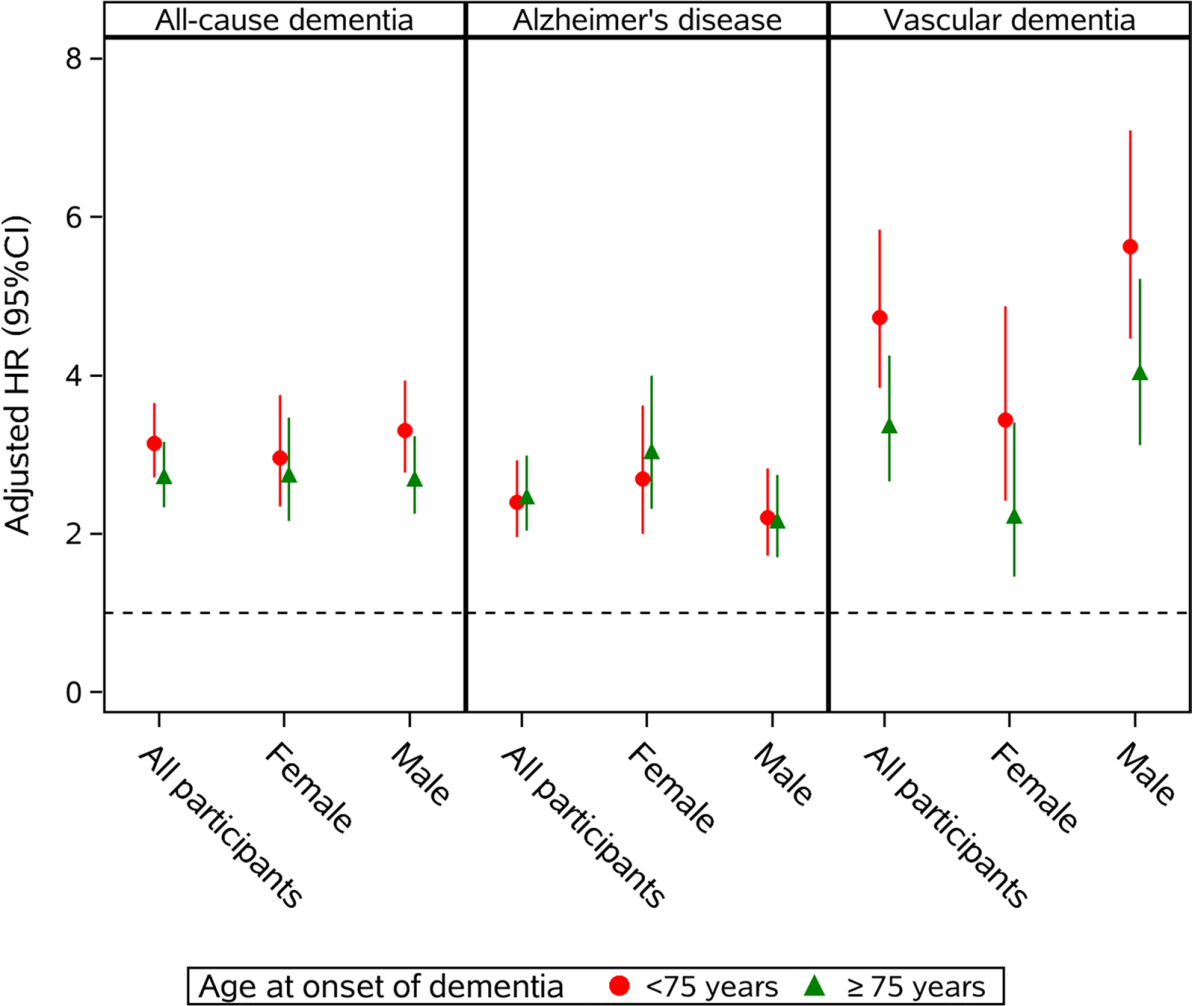
Sex-specific hazard ratios (HRs) on the association of T2DM and dementia subtypes by age at onset of dementia (< 75 and ≥ 75 years). All HRs were adjusted for age at last follow-up, race/ethnicity, educational years, income level, physical activity level, leisure activities, body mass index (BMI), smoking status, hypertension status and APOE 4 allele status

When the associations with age at onset of dementia were analyzed, there was a trend that T2DM had higher effect on all-cause dementia that occurred before age 75 years (3.14, 2.71–3.65) than events that occurred after age 75 years (2.72, 2.33–3.16). Similar trend was found in both male and female. Specific analysis with dementia subtypes showed that the effect of T2DM on AD was not greatly differed by age at onset of dementia, while the effect of diabetes on VD before age 75 was suggested greater than that of VD occurred after age 75 (Fig. 1, Additional file 5: Table S4).

### Timing of T2DM and dementia (Fig. 2 and Additional file 6: Table S5)

**Fig. 2:**
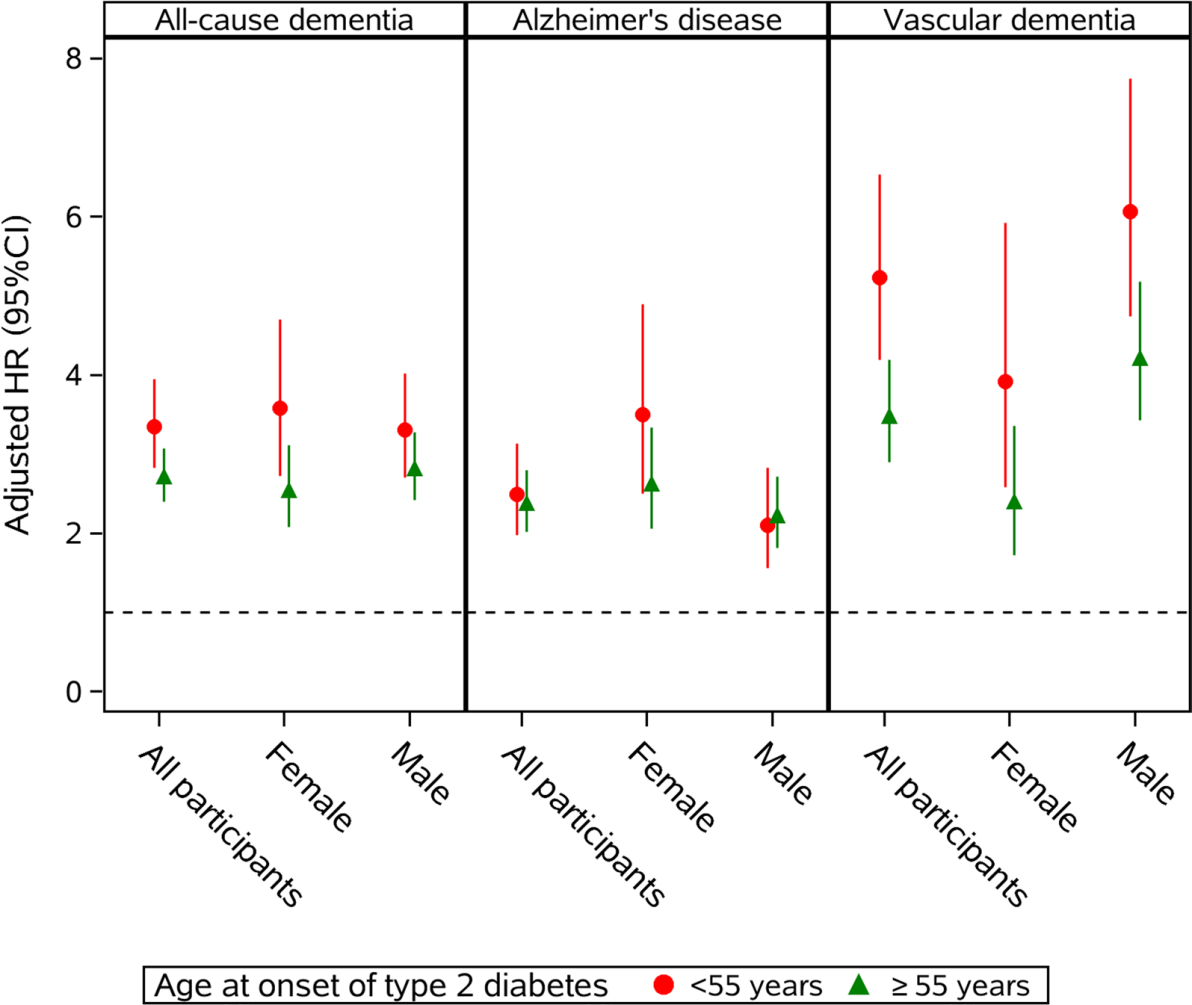
Sex-specific hazard ratios (HRs) on the association of T2DM and dementia subtypes by age at onset of type 2 diabetes (< 55 and ≥ 55 years). All HRs were adjusted for age at last follow-up, race/ethnicity, educational years, income level, physical activity level, leisure activities, body mass index (BMI), smoking status, hypertension status and APOE 4 allele status

When the associations of timing of T2DM and dementia were analyzed, there was a trend that people who experienced T2DM before age 55 had higher risk of all-cause dementia (3.34, 2.82–3.94) than those who had T2DM after age 55 years (2.71, 2.39–3.07). Similar trend was observed in both male and female. Specific analysis with dementia subtypes showed that the elevated effect of early onset T2DM (< 55 years) on dementia was mainly reflected in its association with VD, i.e., experiencing T2DM before age 55 years had suggested higher risk of VD than those with T2DM after 55 years (Fig. 2, Additional file 6: Table S5).

### Insulin use and dementia (Table 3)

**Table 3:** Sex-specific hazard ratios (HRs) between insulin use in type 2 diabetes and dementia subtypes*

	All-cause dementia			Alzheimer's disease			Vascular dementia		
	Dementia events (n)	Events per 1000 person-years	Adjusted HR (95% CI)*	Dementia events (n)	Events per 1000 person-years	Adjusted HR (95% CI)*	Dementia events (n)	Events per 1000 person-years	Adjusted HR (95% CI)*
People with no diabetes at all	2561	0.54	Reference	1918	0.40	Reference	817	0.17	Reference
All patients with type 2 diabetes									
With insulin	153	3.96	4.71 (3.91, 5.66)	82	2.11	3.64 (2.83, 4.68)	94	2.42	7.23 (5.67, 9.24)
Without insulin	409	2.25	2.50 (2.21, 2.82)	240	1.32	2.12 (1.81, 2.48)	208	1.14	3.32 (2.78, 3.97)
Ratio of HR (With/Without insulin)			1.54 (1.00, 2.37)			1.22 (0.67, 2.22)			1.92 (1.06, 3.48)
Female patients with type 2 diabetes									
With insulin	52	3.40	3.64 (2.60, 5.11)	34	2.22	3.75 (2.45, 5.73)	24	1.56	3.80 (2.33, 6.20)
Without insulin	254	3.78	2.28 (1.86, 2.78)	134	1.99	2.44 (1.91, 3.10)	148	2.20	1.97 (1.41, 2.74)
Ratio of HR (With/Without insulin)			1.84 (1.26, 2.68)			1.77 (1.11, 2.81)			2.19 (1.23, 3.88)
Male patients with type 2 diabetes									
With insulin	101	4.32	4.54 (3.52, 5.86)	48	2.05	3.30 (2.30, 4.72)	70	2.99	7.08 (5.09, 9.83)
Without insulin	155	1.35	2.39 (2.05, 2.80)	106	0.92	1.86 (1.51, 2.30)	60	0.52	3.50 (2.82, 4.35)
Ratio of HR (With/Without insulin)			1.73 (1.32, 2.26)			1.60 (1.09, 2.35)			1.91 (1.37, 2.65)

^*^ All HRs were adjusted for age at last follow-up, race/ethnicity, educational years, income level, physical activity level, leisure activities, body mass index (BMI), smoking status, hypertension, APOE4 allele status and HbA1c level

Patients with T2DM using insulin had higher risk of all-cause dementia than those without insulin (RHR 1.54; 95%CI 1.00–2.37). Specific analysis with dementia subtypes showed that compared to people without using insulin, insulin use nearly doubled the risk of VD (RHR 1.92; 95%CI 1.06–3.48).

### Diabetes’ complications and dementia (Tables 4, 5 and Additional file 7: Table S6)

**Table 4:** Hazard ratios (HRs) between type2 diabetes (T2DM) with complications and dementia subtypes in all participants*

	Adjusted HR (95% CI)*		
	All-cause dementia	Alzheimer's disease	Vascular dementia
T2DM + No complications	2.39 (2.11, 2.70)	2.00 (1.71, 2.36)	3.36 (2.81, 4.02)
T2DM + complications	5.18 (4.38, 6.13)	4.19 (3.36, 5.24)	7.22 (5.71, 9.14)
T2DM + No coma	2.88 (2.58, 3.20)	2.39 (2.08, 2.74)	4.02 (3.44, 4.71)
T2DM + coma	9.03 (4.29, 19.02)	7.47 (2.79, 20.00)	16.50 (6.81, 39.96)
T2DM + ketoacidosis	2.87 (2.58, 3.20)	2.38 (2.07, 2.74)	4.03 (3.44, 4.71)
T2DM + No ketoacidosis	5.81 (3.12, 10.84)	5.16 (2.31, 11.49)	9.09 (4.06, 20.37)
T2DM + No renal	2.81 (2.52, 3.14)	2.37 (2.06, 2.73)	3.94 (3.36, 4.62)
T2DM + renal	7.38 (4.95, 10.98)	4.49 (2.40, 8.40)	10.48 (6.24, 17.61)
T2DM + No ophthalmic	2.64 (2.35, 2.97)	2.19 (1.89, 2.55)	3.74 (3.16, 4.43)
T2DM + ophthalmic	4.70 (3.82, 5.79)	3.88 (2.95, 5.10)	6.42 (4.79, 8.59)
T2DM + No neurological	2.70 (2.41, 3.02)	2.30 (1.99, 2.65)	3.71 (3.15, 4.37)
T2DM + neurological	7.32 (5.56, 9.63)	4.84 (3.22, 7.27)	11.75 (8.30, 16.64)
T2DM + No peripheral circulatory	2.77 (2.48, 3.09)	2.35 (2.04, 2.71)	3.84 (3.27, 4.50)
T2DM + peripheral circulatory	7.89 (5.60, 11.12)	4.60 (2.66, 7.97)	12.74 (8.34, 19.45)

^*^ All HRs were adjusted for age at last follow-up, race/ethnicity, educational years, income level, physical activity level, leisure activities, body mass index (BMI), smoking status, hypertension status and APOE 4 allele status. People with no diabetes at all were used as the reference group in all analyses to make the estimates comparable

**Table 5:** Sex-specific hazard ratios (HRs) between the number of complications in patients with type 2 diabetes and dementia subtypes*

	Adjusted HR (95% CI)*		
	All-cause dementia	Alzheimer's disease	Vascular dementia
People with no diabetes at all	Reference	Reference	Reference
All patients with type 2 diabetes			
0	2.45 (2.17, 2.78)	2.03 (1.73, 2.38)	3.48 (2.91, 4.15)
1	4.00 (3.24, 4.94)	3.80 (2.93, 4.93)	4.75 (3.47, 6.49)
≥ 2	8.20 (6.21, 10.83)	5.10 (3.33, 7.81)	12.85 (9.04, 18.25)
Female patients with type 2 diabetes			
0	2.52 (2.08, 3.05)	2.53 (2.00, 3.18)	2.51 (1.85, 3.42)
1	3.65 (2.54, 5.25)	3.76 (2.44, 5.81)	3.44 (1.89, 6.27)
≥ 2	7.59 (4.39, 13.14)	8.06 (4.17, 15.57)	8.63 (3.84, 19.38)
Male patients with type 2 diabetes			
0	2.42 (2.08, 2.81)	1.76 (1.43, 2.16)	4.01 (3.29, 4.89)
1	4.36 (3.39, 5.61)	3.95 (2.88, 5.43)	5.87 (4.11, 8.37)
≥ 2	8.57 (6.24, 11.76)	4.15 (2.40, 7.19)	14.98 (10.24, 21.89)

^*^ All HRs were adjusted for age at last follow-up, race/ethnicity, educational years, income level, physical activity level, leisure activities, body mass index (BMI), smoking status, hypertension status and APOE 4 allele status

Compared to people with no diabetes’ complication, people with complications had doubled risk of all-cause dementia, AD and VD, both in male and female (Table 4, Additional file 7: Table S6). Great number of complications was linked to higher risk of all-cause dementia, AD and VD. The effect of number of complications on risk of AD was greater in women than in men (Table 5). As to specific types of complication, generally, diabetes’ complications had greater effect on AD in women than in men (except for renal complication), and had greater effect on VD in men than in women (except for ketoacidosis) (Additional file 7: Table S6).

## Discussion

### Summary of finding

In this population-based cohort study, we found there was an interaction between sex and T2DM. Women with T2DM are 1.5 times more likely to experience AD than men. There was a trend that T2DM had higher effect on VD that occurred before age 75 years than events that occurred after age 75. In addition, there was a trend that early onset T2DM (defined as younger than age 55 years) was related to higher risk of VD than later onset T2DM (later than age 55 years). Insulin use is associated with increased risk of dementia. Both numbers and types of diabetes’ complications modulate the risk of dementia.

### T2DM and dementia

A few systematic reviews and meta-analysis have examined the association between T2DM and dementia [7, 28]. Zhang et al. found that patients with diabetes were associated with 50% higher risk of AD compared with those without diabetes (RR 1.53, 95%CI 1.42–1.63) [28]. Similarly, another review reported that diabetes was associated with 1.3–1.9 times risk of cognitive impairment and dementia [7]. Consistent with prior studies, we observed that white people who experienced T2DM had 2.85 times higher risk of all-cause dementia, and significant associations were also observed with dementia subtypes. In addition, evidence has shown that younger age at onset of T2DM was associated with higher risk of dementia. Claudio et al. reported at age 70, every 5-year younger age at onset of T2DM was associated with 24% higher risk of all-cause dementia 1.24 (95% CI, 1.06–1.46) [29], while this study did not separate the analysis by dementia subtypes. Our further analysis by timing of T2DM with dementia subtypes showed that experiencing T2DM earlier (younger than 55 years) was related to higher risk of VD than experiencing T2DM later (after 55 years).

### Sex specific association

Whether there is a sex specific association between T2DM and dementia has been unclear. Gong et al. reported no gender differences in the association between diabetes and dementia [11]. However, T2DM was self-reported this study, and some key covariates such as ApoE4 allele status and physical activity were not adjusted. Another study also did not find an interaction between sex and diabetes on global function and cognitive domains [13]. In contrast, a cross-sectional study showed that women with diabetes were 1.29 times more likely to develop dementia than men, but the study did not separate the analysis by dementia subtypes [10]. Another study found that women with diabetes had a higher risk of developing dementia than those without diabetes, while this finding was not observed in men with diabetes [12]. A pooled analysis of 2.3 million people found that diabetes puts women at 19% greater risk of developing VD than men. However, this result was based on the exclusion of data from two large cohorts which together accounted for 96% of all dementia cases [9]. In our findings, although no sex differences between T2DM and all-cause dementia or VD were observed, stratified analysis showed women with T2DM had 50% higher risk of AD than men with T2DM.

### Insulin use and dementia

Previous studies have found insulin use increased the risk of all-cause dementia, while its association with dementia subtypes was unclear. A systematic review and meta-analysis including 144 studies found that T2DM patients who used insulin had 1.36 times higher risk of all-cause dementia than those without insulin therapy [7], while this review did not separate the analysis by sex and dementia subtypes. Another meta-analysis also showed that insulin use was associated with increased risk of AD (HR 1.60; 95%CI 1.13–2.26) compared to those no antidiabetic drug use [19]. In contrast, Weinstein et al. reported that T2DM patients with insulin use was related to 60% higher risk of all-cause dementia compared to those who did not use insulin, while no significant association with AD was observed [20]. Compared to T2DM patients without using insulin, we observed that insulin use was associated with increased risk of all-cause dementia and VD, but no significant association with AD was found. Sex specific analysis showed that insulin use was linked to 2.2 times higher risk of VD in males, while elevated risk was not observed in females.

### Diabetes’ complications and dementia

A cohort study by Exalto et al. has found that T2DM patients who have severe retinal disease had a 42% increased risk of dementia [15]. Po-Yin et al. reported the vascular complications were association with 1.3–3.6 times higher risk of all-cause dementia [16], but this cohort lacked information on some key covariates, including smoking, alcohol consumption, years of education and economic status, so the conclusions obtained might be biased. Consistent with prior studies, we also found that T2DM patients with complications were at increased risk of dementia in both men and women, and the greater the number of complications, the higher risk of developing dementia.

Insulin use, diabetes’ complications and having T2DM at an early age all indicate the severity of diabetes. Our findings also showed that insulin use, great number of diabetes’ complications, and early onset T2DM (before age 55) were all linked to higher risk of all-cause dementia. This implied that the more severe the diabetes, the higher risk of dementia.

### Mechanisms

*Sex difference in the association between T2DM and dementia.* First, some sex-specific risk factors might play a role in the observed sex differences. Gestational diabetes has been associated with higher risks of T2DM and cognitive impairment later in life [30]. Early menopause (either natural or surgical menopause) has been associated with higher risks of cognitive decline, and dementia and 1.20–1.32 times elevated risk of diabetes [31]. These female-specific factors confer excess risk to both diabetes and AD in women. Second, there might be an interaction between sex, cardiovascular and genetic risk factors, which are all related to risk of T2DM and dementia. Hypertension in midlife increased the risk of dementia among women only, although hypertension was more prevalent among men in midlife [32]. In addition, depression and sleep disorders, both risk factors for and T2DM and AD, are also known to be more prevalent in women [33]. Furthermore, sex also modulates the susceptibility to AD conferred by APOE genotype. APOE e4 was associated with a higher risk of AD in females than in males [34]. In addition, our findings also indicated that the development and complications of T2DM may differ by sex, affecting the connection between T2DM and dementia.

*The association between insulin use and dementia.* Insulin use may induce the onset of hypoglycemia, which increases the risk of dementia [35]. Studies have speculated that insulin increased the risk of dementia due to hypoglycaemia [19, 22], and other studies have confirmed that the frequency of hypoglycaemia is related to the duration of insulin use [36, 37]. In addition, patients who take insulin gain weight [38]. Insulin inhibits the lipase action of fat cells, thus reducing the release of free fatty acids. However, free fatty acids can promote Aβ deposition and inhibit Aβ clearance, which is closely related to the mechanism of dementia [18]. Last, insulin is more likely to be used by patients with more severe and prolonged conditions [39]. Chronically high insulin levels can further lead to insulin resistance [18].

### Strengths and limitations

The strengths of this study include the large sample size which enabled us to examine the associations with dementia subtypes and to perform subgroup or stratified analyses. Second, T2DM and dementia were ascertained from primary care, hospital admissions and mortality data, avoiding bias from self-reported data. T2DM and dementia outcomes have been validated in previous studies, demonstrating positive predictive value for all-cause dementia were 80–87% [40]. Furthermore, we also analyzed the effect of T2DM with specific types of complications on dementia, which usually not reported in previous studies. Our study also has several limitations. First, 95% of the involved population were white, which may limit the extrapolation of our findings. Second, we lacked information on duration and dose of insulin use, and the concurrence use of other oral antidiabetic drugs was unclear. Furthermore, there might be some other comorbidities that affect the relationship between T2DM and dementia.

### Perspectives and significance

Our study demonstrated that women with T2DM were 1.56 times more likely to experience AD than men. Early onset T2DM (younger than age 55 years) might be related to higher risk of VD than later onset T2DM (later than age 55 years). Insulin use was linked to 1.5- and 1.9-times higher risk of all-cause dementia and VD, respectively. Different types and numbers of complications modulate the risk of dementia in patients with T2DM. Adopting a sex-sensitive strategy to address the risk of dementia in patients with T2DM is instrumental for a precision medicine approach. Meanwhile, it is warranted to consider patients' age at onset of T2DM, insulin use status and complications conditions.

## Supplementary Information

**Additional file 1: Figure S1.** Flowchart of participants included in the analysis.

**Additional file 2: Table S1.** Codes used in the UK Biobank study to identify variates.

**Additional file 3: Table S2.** Sex-specific hazard ratios (HRs) and 95%CIs between type 2 diabetes and dementia subtypes further adjusted for depressive status.

**Additional file 4: Table S3.** Sex-specific hazard ratios (HRs) and 95%CIs between type 2 diabetes and dementia subtypes further adjusted for CVD status.

**Additional file 5: Table S4.** Sex-specific hazard ratios (HRs) between different types 2 diabetes and dementia subtypes by age of dementia.

**Additional file 6: Table S5.** Sex-specific hazard ratios (HRs) between type 2 diabetes and dementia subtypes by age of diabetes.

**Additional file 7: Table S6.** Sex-specific hazard ratios (HRs) between type2 diabetes (T2DM) with complications and dementia subtypes.

**Additional file 8: Appendix.** STROBE Statement—checklist of items that should be included in reports of cohort studies.

## Data Availability

UK Biobank data are available via www.ukbiobank.ac.uk. Syntax for the generation of derived variables and for the analysis used for this study will be submitted to UK Biobank for record.
