# Supplementary figures and images for: Sex-specific associations between diabetes and dementia: the role of age at onset of disease, insulin use and complications
Source: Biol Sex Differ. 2023 Feb 20;14:9. doi: 10.1186/s13293-023-00491-1 (PMC9940390; doi:10.1186/s13293-023-00491-1)

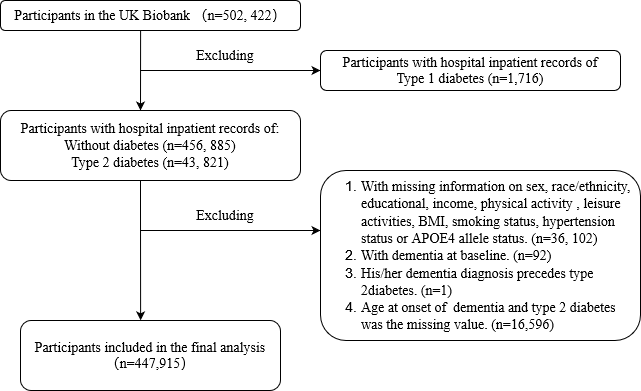


**Figure S1**. Flowchart of participants included in the analysis

Supplement: Supplementary file 1 — Additional file 1: Figure S1. Flowchart of participants included in the analysis. [file 13293_2023_491_MOESM1_ESM.docx]
