# Supplementary material for: Sex-specific associations between diabetes and dementia: the role of age at onset of disease, insulin use and complications
Source: Biol Sex Differ. 2023 Feb 20;14:9. doi: 10.1186/s13293-023-00491-1 (PMC9940390; doi:10.1186/s13293-023-00491-1)
Supplement: Supplementary file 2 — Additional file 2: Table S1. Codes used in the UK Biobank study to identify variates. [file 13293_2023_491_MOESM2_ESM.docx]

| **Table S1** Codes used in the UK Biobank study to identify variates | |  |  |
| --- | --- | --- | --- |
|  | **Field IDs** | **ICD-9** | **ICD-10** |
| **T2DM** |  |  |  |
| Type 2 diabetes |  | 250 | E11 |
| Insulin use | 6153, 6177 |  |  |
| T2DM with coma |  |  | E110 |
| T2DM with ketoacidosis |  |  | E111 |
| T2DM with renal |  |  | E112 |
| T2DM with ophthalmic |  |  | E113 |
| T2DM with neurological |  |  | E114 |
| T2DM with peripheral circulatory |  |  | E115 |
| Age at onset of T2DM | 2976 |  |  |
| **Dementia** |  |  |  |
| All-cause dementia |  | 290.1 | F00, F01, G30 |
| Alzheimer's disease |  | 290.1 | F00, G30 |
| Vascular dementia |  |  | F01 |
| Age at onset of dementia | 130836, 130838, 130840, 42018,  42020, 42024 |  |  |
| **Covariates** |  |  |  |
| Age at last follow up | 21022，21003 |  |  |
| Race/ethnicity | 21000 |  |  |
| Years of education | 6138, 845 |  |  |
| Income level | 738, 26411, 26418, 26428 |  |  |
| Smoking status | 20116 |  |  |
| Physical activity strength | 104910, 884 |  |  |
| Number of leisure activities | 6160 |  |  |
| BMI | 21001 |  |  |
| Hypertension status | 6150, 6153 |  |  |
| APOE4 allele status |  |  |  |
| HbA1c level | 30750 |  |  |
| Cardiovascular diseases (CVD) status |  |  |  |
| heart attack | 6150, 3894 |  |  |
| angina | 6150, 3627 |  |  |
| stroke | 6150, 4056 |  |  |
| Depressive status |  |  | F32 |
