# Supplementary material for: Sex-specific associations between diabetes and dementia: the role of age at onset of disease, insulin use and complications
Source: Biol Sex Differ. 2023 Feb 20;14:9. doi: 10.1186/s13293-023-00491-1 (PMC9940390; doi:10.1186/s13293-023-00491-1)
Supplement: Supplementary file 3 — Additional file 3: Table S2. Sex-specific hazard ratios (HRs) and 95%CIs between type 2 diabetes and dementia subtypes further adjusted for depressive status. [file 13293_2023_491_MOESM3_ESM.docx]

| **Table S2** Sex-specific hazard ratios (HRs) and 95%CIs between type 2 diabetes and dementia subtypes further adjusted for depressive status* | | | | | | | | | | | |
| --- | --- | --- | --- | --- | --- | --- | --- | --- | --- | --- | --- |
|  | **All-cause dementia** | | |  | **Alzheimer's disease** | | |  | **Vascular Dementia** | | |
|  | **Dementia events (n)** | **Events per 1000 person-years** | **Adjusted HR (95% CI)*** |  | **Dementia events (n)** | **Events per 1000 person-years** | **Adjusted HR**  **(95% CI)*** |  | **Dementia events (n)** | **Events per 1000 person-years** | **Adjusted HR**  **(95% CI)*** |
| People with no diabetes at all | 2561 | 0.54 | Reference |  | 1918 | 0.40 | Reference |  | 817 | 0.17 | Reference |
| People with type 2 diabetes |  |  |  |  |  |  |  |  |  |  |  |
| All patients | 562 | 2.55 | 2.68 (2.41, 2.98) |  | 322 | 1.46 | 2.26 (1.96, 2.59) |  | 302 | 1.37 | 3.65 (3.12, 4.27) |
| Female patients | 207 | 2.51 | 2.38 (2.02, 2.82) |  | 140 | 1.69 | 2.45 (2.00, 2.99) |  | 84 | 1.02 | 2.31 (1.76, 3.02) |
| Male patients | 355 | 2.57 | 2.87 (2.53, 3.26) |  | 182 | 1.31 | 2.12 (1.78, 2.52) |  | 218 | 1.57 | 4.60 (3.87, 5.46) |
| Ratio of HR (Female/Male) |  |  | 1.13 (0.92, 1.39) |  |  |  | 1.48 (1.14, 1.92) |  |  |  | 0.84 (0.61, 1.16) |
| * All HRs were adjusted for age at last follow up, race/ethnicity, educational years, income level, physical activity level, leisure activities, body mass index (BMI), smoking status, hypertension status APOE4 allele status and depressive status. | | | | | | | | | | | |
