# Supplementary material for: Sex-specific associations between diabetes and dementia: the role of age at onset of disease, insulin use and complications
Source: Biol Sex Differ. 2023 Feb 20;14:9. doi: 10.1186/s13293-023-00491-1 (PMC9940390; doi:10.1186/s13293-023-00491-1)
Supplement: Supplementary file 4 — Additional file 4: Table S3. Sex-specific hazard ratios (HRs) and 95%CIs between type 2 diabetes and dementia subtypes further adjusted for CVD status. [file 13293_2023_491_MOESM4_ESM.docx]

| **Table S3** Sex-specific hazard ratios (HRs) and 95%CIs between type 2 diabetes and dementia subtypes further adjusted for CVD status* | | | | | | | |  |  |  |  |
| --- | --- | --- | --- | --- | --- | --- | --- | --- | --- | --- | --- |
|  | **All-cause dementia** | | |  | **Alzheimer's disease** | | |  | **Vascular Dementia** | | |
|  | **Dementia events (n)** | **Events per 1000 person-years** | **Adjusted HR**  **(95% CI)*** |  | **Dementia events (n)** | **Events per 1000 person-years** | **Adjusted HR**  **(95% CI)*** |  | **Dementia events (n)** | **Events per 1000 person-years** | **Adjusted HR**  **(95% CI)*** |
| People with no diabetes at all | 2561 | 0.54 | Reference |  | 1918 | 0.40 | Reference |  | 817 | 0.17 | Reference |
| People with type 2 diabetes |  |  |  |  |  |  |  |  |  |  |  |
| All patients | 562 | 2.55 | 2.58 (2.31, 2.87) |  | 322 | 1.46 | 2.23 (1.94, 2.56) |  | 302 | 1.37 | 3.35 (2.86, 3.92) |
| Female patients | 207 | 2.51 | 2.59 (2.19, 3.06) |  | 140 | 1.69 | 2.68 (2.19, 3.27) |  | 84 | 1.02 | 2.50 (1.91, 3.26) |
| Male patients | 355 | 2.57 | 2.58 (2.27, 2.93) |  | 182 | 1.31 | 1.97 (1.66, 2.35) |  | 218 | 1.57 | 3.87 (3.25, 4.61) |
| Ratio of HR (Female/Male) |  |  | 1.23 (1.00, 1.51) |  |  |  | 1.60 (1.23, 2.08) |  |  |  | 0.93 (0.67, 1.28) |
| * All HRs were adjusted for age at last follow up, race/ethnicity, educational years, income level, physical activity level, leisure activities, body mass index (BMI), smoking status, hypertension status APOE4 allele status and CVD status. | | | | | | | | | | | |
