# Supplementary material for: Sex-specific associations between diabetes and dementia: the role of age at onset of disease, insulin use and complications
Source: Biol Sex Differ. 2023 Feb 20;14:9. doi: 10.1186/s13293-023-00491-1 (PMC9940390; doi:10.1186/s13293-023-00491-1)
Supplement: Supplementary file 5 — Additional file 5: Table S4. Sex-specific hazard ratios (HRs) between different types 2 diabetes and dementia subtypes by age of dementia. [file 13293_2023_491_MOESM5_ESM.docx]

| **Table S4** Sex- specific hazard ratios (HRs) between different types 2 diabetes and dementia subtypes by age of dementia* | | | | | | | |  |
| --- | --- | --- | --- | --- | --- | --- | --- | --- |
|  | **All-cause dementia** | |  | **Alzheimer's disease** | |  | **Vascular Dementia** | |
|  | **<75 years** | **≥75 years** |  | **<75 years** | **≥75 years** |  | **<75 years** | **≥75 years** |
| People with no diabetes at all | Reference | Reference |  | Reference | Reference |  | Reference | Reference |
| People with type 2 diabetes |  |  |  |  |  |  |  |  |
| All patients | 3.14 (2.71, 3.65) | 2.72 (2.33, 3.16) |  | 2.40 (1.96, 2.93) | 2.47 (2.04, 2.99) |  | 4.73 (3.84, 5.84) | 3.36 (2.66, 4.25) |
| Female patients | 2.96 (2.34, 3.75) | 2.74 (2.16, 3.46) |  | 2.69 (2.00, 3.62) | 3.04 (2.31, 4.00) |  | 3.43 (2.42, 4.87) | 2.22 (1.46, 3.40) |
| Male patients | 3.30 (2.77, 3.93) | 2.69 (2.25, 3.23) |  | 2.20 (1.72, 2.82) | 2.16 (1.70, 2.74) |  | 5.63 (4.46, 7.09) | 4.04 (3.12, 5.22) |
| * All HRs were adjusted for age at last follow up, race/ethnicity, educational years, income level, physical activity level, leisure activities, body mass index (BMI), smoking status, hypertension status and APOE4 allele status. | | | | | | | | |
