# Supplementary material for: Sex-specific associations between diabetes and dementia: the role of age at onset of disease, insulin use and complications
Source: Biol Sex Differ. 2023 Feb 20;14:9. doi: 10.1186/s13293-023-00491-1 (PMC9940390; doi:10.1186/s13293-023-00491-1)
Supplement: Supplementary file 6 — Additional file 6: Table S5. Sex-specific hazard ratios (HRs) between type 2 diabetes and dementia subtypes by age of diabetes. [file 13293_2023_491_MOESM6_ESM.docx]

| **Table S5** Sex- specific hazard ratios (HRs) between type 2 diabetes and dementia subtypes by age of diabetes* | | | |
| --- | --- | --- | --- |
|  | **Adjusted HR (95% CI)*** | | |
|  | **All-cause dementia** | **Alzheimer's disease** | **Vascular Dementia** |
| People with no diabetes at all | Reference | Reference | Reference |
| Aged <55 years at first onset of type 2 diabetes |  |  |  |
| All patients | 3.34 (2.82, 3.94) | 2.49 (1.98, 3.13) | 5.23 (4.19, 6.53) |
| Female patients | 3.58 (2.72, 4.70) | 3.50 (2.50, 4.89) | 3.91 (2.58, 5.92) |
| Male patients | 3.30 (2.70, 4.02) | 2.10 (1.56, 2.82) | 6.06 (4.74, 7.75) |
| Aged ≥55 years at first onset of type 2 diabetes |  |  |  |
| All patients | 2.71 (2.39, 3.07) | 2.37 (2.02, 2.79) | 3.48 (2.89, 4.19) |
| Female patients | 2.54 (2.08, 3.11) | 2.62 (2.06, 3.33) | 2.40 (1.72, 3.35) |
| Male patients | 2.81 (2.42, 3.27) | 2.22 (1.81, 2.71) | 4.21 (3.42, 5.18) |
| * All HRs were adjusted for age at last follow up, race/ethnicity, educational years, income level, physical activity level, leisure activities, body mass index (BMI), smoking status, hypertension status and APOE4 allele status. | | | |
