# Supplementary material for: Sex-specific associations between diabetes and dementia: the role of age at onset of disease, insulin use and complications
Source: Biol Sex Differ. 2023 Feb 20;14:9. doi: 10.1186/s13293-023-00491-1 (PMC9940390; doi:10.1186/s13293-023-00491-1)
Supplement: Supplementary file 7 — Additional file 7: Table S6. Sex-specific hazard ratios (HRs) between type2 diabetes (T2DM) with complications and dementia subtypes. [file 13293_2023_491_MOESM7_ESM.docx]

| **Table S6** Sex- specific hazard ratios (HRs) between type2 diabetes (T2DM) with complications and dementia subtypes * | | | | | | | | |
| --- | --- | --- | --- | --- | --- | --- | --- | --- |
|  | **All-cause dementia** | |  | **Alzheimer's disease** | |  | **Vascular Dementia** | |
|  | **Female** | **Male** |  | **Female** | **Male** |  | **Female** | **Male** |
| T2DM+ No complications | 2.49 (2.05, 3.02) | 2.33 (2.01, 2.72) |  | 2.48 (1.96, 3.13) | 1.75 (1.42, 2.15) |  | 2.47 (1.81, 3.37) | 3.86 (3.15, 4.72) |
| T2DM+ complications | 4.38 (3.25, 5.91) | 5.65 (4.65, 6.87) |  | 4.63 (3.25, 6.61) | 4.02 (3.05, 5.29) |  | 4.47 (2.78, 7.18) | 8.70 (6.71, 11.28) |
| T2DM+ No coma | 2.82 (2.38, 3.33) | 2.92 (2.58, 3.32) |  | 2.87 (2.35, 3.51) | 2.13 (1.79, 2.54) |  | 2.79 (2.13, 3.65) | 4.72 (3.97, 5.61) |
| T2DM+ coma | 4.48 (0.63, 31.85) | 10.86 (4.86, 24.28) |  |  | 10.24 (3.82, 27.47) |  | 10.94 (1.54, 77.92) | 18.89 (7.03, 50.75) |
| T2DM+ No ketoacidosis | 2.75 (2.32, 3.26) | 2.96 (2.60, 3.35) |  | 2.78 (2.27, 3.42) | 2.17 (1.82, 2.58) |  | 2.71 (2.06, 3.56) | 4.76 (4.01, 5.66) |
| T2DM+ ketoacidosis | 8.99 (4.04, 20.01) | 3.83 (1.43, 10.22) |  | 8.56 (3.21, 22.84) | 2.87 (0.72, 11.49) |  | 11.97 (3.84, 37.28) | 7.33 (2.35, 22.85) |
| T2DM+ No renal | 2.81 (2.37, 3.32) | 2.83 (2.49, 3.22) |  | 2.88 (2.36, 3.52) | 2.10 (1.76, 2.50) |  | 2.74 (2.08, 3.61) | 4.62 (3.88, 5.50) |
| T2DM+ renal | 4.25 (1.59, 11.37) | 8.81 (5.71, 13.59) |  | 1.63 (0.23, 11.60) | 5.75 (2.98, 11.11) |  | 7.52 (2.41, 23.49) | 11.87 (6.66, 21.13) |
| T2DM+ No ophthalmic | 2.56 (2.13, 3.08) | 2.70 (2.35, 3.10) |  | 2.60 (2.08, 3.25) | 1.98 (1.64, 2.39) |  | 2.52 (1.86, 3.39) | 4.42 (3.67, 5.33) |
| T2DM+ ophthalmic | 4.61 (3.27, 6.49) | 4.81 (3.73, 6.19) |  | 4.61 (3.04, 6.99) | 3.53 (2.48, 5.03) |  | 4.86 (2.85, 8.30) | 7.33 (5.24, 10.27) |
| T2DM+ No neurological | 2.74 (2.31, 3.25) | 2.69 (2.35, 3.07) |  | 2.75 (2.24, 3.39) | 2.06 (1.72, 2.46) |  | 2.71 (2.05, 3.58) | 4.28 (3.56, 5.13) |
| T2DM+ neurological | 5.21 (2.87, 9.45) | 8.21 (6.05, 11.14) |  | 5.78 (2.87, 11.61) | 4.51 (2.74, 7.40) |  | 5.89 (2.43, 14.25) | 14.12 (9.72, 20.51) |
| T2DM+ No peripheral circulatory | 2.76 (2.33, 3.28) | 2.79 (2.45, 3.18) |  | 2.79 (2.28, 3.43) | 2.12 (1.78, 2.53) |  | 2.78 (2.12, 3.65) | 4.44 (3.71, 5.31) |
| T2DM+ peripheral circulatory | 7.17 (3.21, 16.01) | 8.10 (5.56, 11.80) |  | 7.52 (2.82, 20.07) | 3.94 (2.04, 7.60) |  | 5.84 (1.45, 23.46) | 14.40 (9.26, 22.40) |
| * All HRs were adjusted for age at last follow up, race/ethnicity, educational years, income level, physical activity level, leisure activities, body mass index (BMI), smoking status, hypertension status and APOE4 allele status. People with no diabetes at all were used as the reference group in all analyses to make the estimates comparable. | | | | | | | | |
