# Supplementary material for: Sex-specific associations between diabetes and dementia: the role of age at onset of disease, insulin use and complications
Source: Biol Sex Differ. 2023 Feb 20;14:9. doi: 10.1186/s13293-023-00491-1 (PMC9940390; doi:10.1186/s13293-023-00491-1)
Supplement: Supplementary file 8 — Additional file 8: Appendix. STROBE Statement—checklist of items that should be included in reports of cohort studies. [file 13293_2023_491_MOESM8_ESM.docx]

**STROBE Statement—checklist of items that should be included in reports of cohort studies**

|  | | Item No | Recommendation |  | Relevant content in this paper |
| --- | --- | --- | --- | --- | --- |
| **Title and abstract** | | 1 | (*a*) Indicate the study’s design with a commonly used term in the title or the abstract |  | In Abstract: This prospective study analyzed data of 447 931 participants from the UK Biobank’. |
|  |  |  | (*b*) Provide in the abstract an informative and balanced summary of what was done and what was found |  | In the Methods and findings section of Abstract. |
| Introduction | | | |  |  |
| Background/rationale | | 2 | Explain the scientific background and rationale for the investigation being reported |  | Paragraph 1 and 2 in Introduction |
| Objectives | | 3 | State specific objectives, including any prespecified hypotheses |  | Paragraph 3 in Introduction |
| Methods | | | |  |  |
| Study design | | 4 | Present key elements of study design early in the paper |  | ‘**Participants**’ section in Methods part: ‘The UK Biobank is a large population-based prospective cohort study that recruited over 502 000 participants aged 40-70 years from 2006 to 2010’. |
| Setting | | 5 | Describe the setting, locations, and relevant dates, including periods of recruitment, exposure, follow-up, and data collection |  | ‘**Participants**’ section in Methods part: ‘Written informed consent was obtained for collection of questionnaire and biological data. All participants were linked to hospital data and national death registries from England, Scotland and Wales to determine the date of the first diagnosis of dementia after the baseline assessment.’ |
| Participants | | 6 | (*a*) Give the eligibility criteria, and the sources and methods of selection of participants. Describe methods of follow-up |  | ‘**Participants**’ section in Methods part: ‘A prospective design was adopted based on participants with no dementia at baseline, and if a participant had dementia during follow-up and also experienced T2DM, his/her diagnosis of T2DM had to be in advance of dementia.’. |
|  |  |  | (*b*) For matched studies, give matching criteria and number of exposed and unexposed |  | \| Not applicable (the current study is not a matched study). \| \| --- \| |
| Variables | | 7 | Clearly define all outcomes, exposures, predictors, potential confounders, and effect modifiers. Give diagnostic criteria, if applicable |  | These are defined and presented in the ‘**Exposure and outcome variables**’ and ‘**Covariates**’ sections in Methods part.  **The exposure** was the occurrence of T2DM. Physician-diagnosed T2DM was ascertained from linkage data to primary care, hospital admission and death register records. The International Classification of Diseases 10th Edition (ICD-10) code E11 and ICD-9 code 250 were used to identify participants with T2DM. T2DM-related complication types were defined by the ICD-10 codes E11·0-E11·5 and ICD-9 code 250. Insulin use status was self-reported.  **The outcome** was incident all-cause dementia, including dementia subtypes of AD and VD. The ICD-10 codes F00, F01, G30, and ICD-9 code 290·1 were used to identify participants with all-cause dementia if one or more of these codes were recorded as a primary or secondary diagnosis in the health records. Incident AD was defined by ICD-10 codes F00, G30 and ICD-9 code 290·1. Incident VD was defined by ICD-10 code F01.  **The covariates** included age at last follow up, race/ethnicity, years of education, income level, smoking status, physical activity strength, number of leisure activities, BMI, hypertension status, APOE4 allele status, HbA1c level, cardiovascular diseases (CVD) status and depressive status. |
| Data sources/ measurement | | 8* | For each variable of interest, give sources of data and details of methods of assessment (measurement). Describe comparability of assessment methods if there is more than one group |  | These are presented in the ‘**Exposure and outcome variables**’ section of Methods part. Outcome adjudication for incident dementia was conducted by the UK Biobank Outcome Adjudication team. Physician-diagnosed T2DM and dementia was ascertained from linkage data to primary care, hospital admission and death register records. |
| Bias | | 9 | Describe any efforts to address potential sources of bias |  | In the ‘**Statistical analyses**’ section of Methods part.  The female-to-male risk ratio (RHR) for each type of dementia and T2DM was further adjusted for CVD status and depressive status. |
| Study size | | 10 | Explain how the study size was arrived at |  | ‘**Participants**’ section in Methods part. |
| Quantitative variables | | 11 | Explain how quantitative variables were handled in the analyses. If applicable, describe which groupings were chosen and why |  | ‘**Covariates**’ sections of Methods part. |
| Statistical methods | | 12 | (*a*) Describe all statistical methods, including those used to control for confounding |  | In the ‘**Statistical analyses**’ section of Methods part. We used Cox proportional hazards models to estimate hazard ratios and 95% confidence intervals (HR, 95% CI) while adjusting series of covariates. |
|  |  |  | (*b*) Describe any methods used to examine subgroups and interactions |  | In the ‘**Statistical analyses**’ section of Methods part.  Cox proportional hazards regression models were used to estimate the sex-specific hazard ratios (HR) and 95% confidence intervals (CI) between T2DM and dementia (including all-cause dementia, AD and VD). The interaction term between T2DM and sex was used to obtain the women-to-men ratio of hazard ratios (RHR) for each dementia type and T2DM. |
|  |  |  | (*c*) Explain how missing data were addressed |  | Analyses were based on complete cases. |
|  |  |  | (*d*) If applicable, explain how loss to follow-up was addressed |  | NA |
|  |  |  | (*e*) Describe any sensitivity analyses |  | NA |
| Results | | | |  |  |
| Participants | 13* | (a) Report numbers of individuals at each stage of study—eg numbers potentially eligible, examined for eligibility, confirmed eligible, included in the study, completing follow-up, and analysed | |  | In the ‘**Participants**’ section of Methods part |
|  |  | (b) Give reasons for non-participation at each stage | |  | NA |
|  |  | (c) Consider use of a flow diagram | |  | Supplementary Figure S1 |
| Descriptive data | 14* | (a) Give characteristics of study participants (eg demographic, clinical, social) and information on exposures and potential confounders | |  | Table 1 |
|  |  | (b) Indicate number of participants with missing data for each variable of interest | |  | Table 1 |
|  |  | (c) Summarise follow-up time (eg, average and total amount) | |  | Paragraph 1 in Results: ‘The median (Q1, Q3) follow-up was 11.0 (11.0, 12.0) years.’ |
| Outcome data | 15* | Report numbers of outcome events or summary measures over time | |  | Paragraph 1 in Results. |
| Main results | 16 | (*a*) Give unadjusted estimates and, if applicable, confounder-adjusted estimates and their precision (eg, 95% confidence interval). Make clear which confounders were adjusted for and why they were included | |  | Paragraph 2-6 in Results part. |
|  |  | (*b*) Report category boundaries when continuous variables were categorized | |  | Table 1 |
|  |  | (*c*) If relevant, consider translating estimates of relative risk into absolute risk for a meaningful time period | |  | Table 2-3 and supplementary Table 2-3 |
| Other analyses | 17 | Report other analyses done—eg analyses of subgroups and interactions, and sensitivity analyses | |  |  |
| Discussion | | | |  |  |
| Key results | 18 | Summarise key results with reference to study objectives | |  | In the ‘**Summary of findings**’ section, ‘**T2DM and dementia**’ section, ‘**Sex specific association**’ section and ‘**Insulin use and dementia**’ section and ‘**Diabetes’ complications and dementia**’ section of Discussion part. |
| Limitations | 19 | Discuss limitations of the study, taking into account sources of potential bias or imprecision. Discuss both direction and magnitude of any potential bias | |  | In the ‘**Strengths and limitations**’ section of Discussions part. |
| Interpretation | 20 | Give a cautious overall interpretation of results considering objectives, limitations, multiplicity of analyses, results from similar studies, and other relevant evidence | |  | In the ‘**Conclusion**’ section |
| Generalisability | 21 | Discuss the generalisability (external validity) of the study results | |  | In the ‘**Limitations**’ section: ‘First, the participants were predominantly white, which may limit the extrapolation of the results’. |
| Other information | | | |  |  |
| Funding | 22 | Give the source of funding and the role of the funders for the present study and, if applicable, for the original study on which the present article is based | |  | In the ‘**Funding**’ part of the paper. |

*Give information separately for exposed and unexposed groups.
